# In-Home Respite Care Services Available to Families With Palliative Care Needs in Quebec: Novel Digital Environmental Scan

**DOI:** 10.2196/53078

**Published:** 2024-04-16

**Authors:** Aimee Castro, Gabrielle Lalonde-LeBlond, Zelda Freitas, Antonia Arnaert, Vasiliki Bitzas, John Kildea, Karyn Moffatt, Devon Phillips, Lorne Wiseblatt, Audrey-Jane Hall, Véronique Després, Argerie Tsimicalis

**Affiliations:** 1 Ingram School of Nursing McGill University Montreal, QC Canada; 2 Professional Practice Directorate of Rehabilitation and Multidisciplinary Services CIUSSS West-Central Montreal Montreal, QC Canada; 3 School of Social Work McGill University Montreal, QC Canada; 4 Geriatrics and Palliative Care CIUSSS West-Central Montreal Montreal, QC Canada; 5 Medical Physics Unit McGill University Montreal, QC Canada; 6 School of Information Studies McGill University Montreal, QC Canada; 7 Palliative Care McGill McGill University Montreal, QC Canada; 8 McGill University Health Centre Montreal, QC Canada; 9 Palliative Home-Care Society of Greater Montreal Montreal, QC Canada; 10 St-Raphaël Palliative Care Home and Day Centre Montreal, QC Canada; 11 Nursing Research Shriners Hospitals for Children-Canada Montreal, QC Canada

**Keywords:** respite care, palliative care, caregiving, environmental scan, digital methodology, accessibility

## Abstract

**Background:**

Caregiving dyads in palliative care are confronted with complex care needs. Respite care services can be highly beneficial in alleviating the caregiving burden, supporting survivorship and dying at home. Yet, respite care services are difficult to locate and access in the province of Quebec, Canada, particularly when navigating ubiquitous sources of online health information of varying quality.

**Objective:**

This project aimed to (1) compile a list of at-home palliative respite care services in Quebec, Canada; (2) describe key accessibility features for each respite care service; (3) identify accessibility gaps and opportunities; and (4) describe a novel method for conducting environmental scans using internet search engines, internet-based community health databases, and member checking.

**Methods:**

A novel environmental scan methodology using 2 internet-based targeted databases and 1 internet search engine was conducted. Results were screened and data were extracted, descriptively analyzed, and geographically schematized.

**Results:**

A total of 401 services were screened, and 52 at-home respite care services specific to palliative populations were identified, compiled, and analyzed. These respite care services were characterized by various types of assistance, providers, fees, and serviced geographical regions. Accessibility was explored through the lens of service amenability, availability, eligibility, and compatibility. The data revealed important barriers to accessing respite care services, such as a lack of readily available information on service characteristics, limited availability, and a time-consuming, technical search process for potential respite care users and clinicians to identify appropriate services.

**Conclusions:**

Both methodological and contextual knowledge have been gained through this environmental scan. Few methodologies for conducting internet-based environmental scans have been clearly articulated, so we applied several learnings from other scans and devised a methodology for conducting an environmental scan using the mixed methods of internet search engines, internet-based community health databases, and member checking. We have carefully reported our methods, so that others conducting community health environmental scans may replicate our process. Furthermore, through this scan, we identified assorted respite care services and pinpointed needs in the provision of these services. The findings highlighted that more easily accessible and centralized information about respite care services is needed in Quebec. The data will enable the creation of a user-friendly tool to share with community support services across Quebec and ultimately help alleviate the added burden caregivers and clinicians face when looking for respite care services in fragmented and complex digital spaces.

## Introduction

### Overview

Palliative caregiving is a particularly intensive form of caregiving. Respite care is one of the essential services helping to support informal caregivers (ie, generally individuals with a preexisting relationship to the care recipient, with no additional training, and contributing unpaid work), and care recipients, particularly those in the palliative stage of care [1]. The goal of respite care is to provide short-term relief to informal caregivers and care recipients from their dyadic care-giving and care-receiving relationship by allowing both parties to spend time away from each other, interact with others, and perform activities that they enjoy or need to do [2-4]. During respite, another person acts as the care recipient’s temporary caregiver [2,4]. Respite can be accessed via a variety of service provisions (eg, palliative care, hospice day centers, and home care), offered in different settings (at home, in a facility, in the hospital, and in the community) and provided by an array of health care personnel to individuals coping with disabilities or illnesses [2,3,5,6].

For dyads in a palliative stage of care, respite care often contributes to supporting death in the home setting, which most patients prefer, all the while improving both parties’ psychosocial well-being and quality of life [2,6-9]. Additionally, these services are linked to decreased hospital admissions, health care costs, and use of aggressive care at the end of life [8,10]. In fact, dyads coping with terminal illnesses and needing palliative care support are increasingly requesting respite care services in Canada [3,10,11]. Despite these benefits, there seems to be no clear, comprehensive, and easily accessible information on overall or specific resources offered in Quebec.

In Canada, and particularly in Quebec, the provision of respite care falls outside the Canadian Health Act, which governs health care provision across Canada. As a result, a patchwork of services, funded through a variety of public, private-for-profit, and private nonprofit initiatives, is offered to nearly 1.5 million informal caregivers and care recipients in Quebec [12]. Government guides direct caregivers to their local Centres intégrés de santé et de services sociaux (in English: integrated health and social services centers) and Centres intégrés universitaires de santé et de services sociaux (in English: integrated university health and social services centers) for details on respite care services as opposed to specific agencies [13]. Additionally, some nonprofit organizations offer web portals for searching respite care services within their target population, such as Portail Répit for caregivers of children living with disabilities. The lack of a seamless respite care access pathway results in a lengthy, multistep process to access services—a process that can be overwhelming for exhausted palliative care dyads, and time-consuming for nurses, who typically oversee respite care service coordination and home care service provision.

Difficulties encountered while searching, locating, and accessing respite care impose an additional, undesirable burden on informal caregivers seeking respite [11,14]. Even with internet access at home, nearly a quarter of Canadians, particularly those most likely to resort to at-home health care services, have very limited internet use and digital skills [15-17]. Consequently, individuals with varying levels of digital literacy (ie, the ability to successfully use and navigate the internet and the associated apps or devices), are stranded to identify a search strategy by themselves [17]. The paucity of relevant information and difficulties in finding available services specific to individual needs may render respite care services inaccessible [7,8,18-20]. Considering the overall preference for death at home, and challenges associated with palliative caregiving, addressing access to information and support services, such as respite care, is essential to ensure all parties are supported through this phase of care.

### Objectives

This environmental scan study aimed to identify and describe the characteristics of in-home respite care services currently available to caregiving dyads with palliative care needs in the French-speaking province of Quebec, by (1) mapping a current list of in-home palliative respite care services available to adults in Quebec; (2) describing and analyzing key offerings and accessibility features for each service; (3) identifying gaps and opportunities to increase accessibility and usage of these services; and (4) describing a methodology for conducting environmental scans using various internet-based sources and member checking.

## Methods

### Overview

Environmental scans methodologically support the systematic collection and analysis of information and services available within a specific environment for addressing the needs of a specific population. While no standard approach exists, this design often relies on searching beyond the academically published literature to identify all currently available programs [21-25]. Environmental scan strategies consist of combining sources of information consolidated from grey literature, internet search engines, and stakeholder consultations to identify all up-to-date and accessible services of a specific type available in a given geographic region [21]. This project implemented a novel environmental scan methodology to compile existing respite care services for individuals with palliative care needs in Quebec.

The novel and iterative strategy we developed consisted of (1) conducting a comprehensive search of internet-based respite and health care databases and internet search engines, (2) identifying and screening results for eligibility, (3) extracting and compiling the data, (4) seeking expert consultations, (5) analyzing the data, and (6) synthesizing the results into a coherent report on respite care services in Quebec. The general framework for scoping reviews by Peters et al [26], was taken into consideration, as were the methods used by related environmental scans, which tended to use analog paper sources and grey literature, rather than digital and internet-based resources [23,27].

### Ethical Considerations

As this was a grey literature–based study and no human or animal participants were involved, ethics approval was not required.

### Eligibility Criteria

The eligibility of respite care services was determined through a 2-step process. First, for respite care services to be considered eligible for this environmental scan, they had to be (1) offered in Quebec, (2) coordinated by an official organization, (3) offered in-person, and (4) offered as a stand-alone service. Home support services that did not specifically mention the concept of respite were excluded, along with Google ads. Remote respite care services (eg, video camera “nanny cams”) and informal respite care provided by family, friends, or self-employed individuals were not considered, as well as services only available when participating in the organization’s broader activities [1].

Second, eligible respite care services were further screened to identify a subgroup of services that (1) were offered in the family’s home and (2) indicated that services were either destined for a population in palliative care or at the end of life or that specialized services for persons in palliative care or at the end of life were offered in conjunction with general respite care.

### Internet-Based Search of Respite Care Services

Respite care services were identified by (1) searching internet-based respite and health care databases and (2) searching the most commonly used internet search engine. The search strategies and methodology were created in collaboration with an expert librarian and reviewed by consulting coauthors to ensure that the keywords used were most appropriate for the Quebec context. Examples of keywords used in these search strategies included “respite care,” “short-term care,” and “home caregiving” (see Table 1 for the full list of keywords).

**Table 1:** Keywords related to the main research question and concept of respite care; keywords were translated from English to French by a bilingual member of the research team, with the corroborating assistance of DeepL Translator (DeepL SE) [28].

Original English keywords	Translated French keywords
Respite care	Soins de répit; service de répit; soins de relève; service de relève
Respite	Répit; relève
Short-term care; short term care	Soins à court terme; soins de courte durée
Sitting service	Service de garde
Adult day-care; adult day care; adult daycare	Soins de jour pour adulte
Day respite facility	Établissement de répit de jour; centre de répit de jour; maison de répit de jour
Hospice at home; home-based palliative care; home hospice	Soins palliatifs à domicile
Hospice day centre; palliative day centre	Centre de jour de soins palliatifs; centre de jour palliatif
Home care; Homecare; home caregiving	Soins à domicile; assistance à domicile
Caregiving help	Aide aux proches aidants; aide aux aidants
Help for caregivers	Aide pour proches aidants; aide pour aidants

### Step 1: Searching Internet-Based Respite and Health Care Databases

#### Overview

A bilingual, French and English, search was conducted using web resource databases intended for caregivers and patients and that are relevant to the subject of caregiving support and respite care in Quebec—the Canadian Cancer Society Community Services Locator and the resource directory for L’Appui Proche Aidants, an organization supporting informal caregiving in Quebec [29,30]. Our search strategy slightly differed from 1 database to the next due to their unique search functionalities.

#### Canadian Cancer Society Community Services Locator

This database was searched using the keywords found in Textbox 1, with Quebec, Canada, listed as the location. No specific search parameters or limitations were applied, and the results were sorted by relevance.

**Search permutation for Google search; “Keyword” was replaced by each keyword listed in Table 1. Quebec, Montreal, Sherbrooke, Trois-Riviere, Chicoutimi, Saint-Jerome, and Saint-Jean-sur-Richelieu were selected due to being populous regions in the province of Quebec.**

For English keywords: “Keyword” AND (“palliative” OR “hospice” OR “dying” OR “end-of-life”) AND (Quebec OR Montreal OR Sherbrooke OR Trois-Rivieres OR Chicoutimi OR Saint-Jerome OR Saint-Jean-sur-Richelieu)

For French keywords: “Keyword” AND (“palliatif” OR “mourant” OR “mourir” OR “fin de vie”) AND (Québec OR Montréal OR Sherbrooke OR Trois-Rivières OR Chicoutimi OR Saint-Jérôme OR Saint-Jean-sur-Richelieu)

#### L’Appui Resource Directory

This database was searched using the “Search by Service” function along with selecting the subcategory listed in the database filters of “respite care services offered in the home.” This directory does not allow for a province-wide search. Thus, the most populated postal codes for each of Quebec’s 18 health regions were used to facilitate the search for services across Quebec [31,32]. The results were automatically sorted from closest to farthest away from the postal code.

### Step 2: Searching an Internet Search Engine

Google, the most popular search engine option in Canada, was used on a private browsing window to further identify respite care services [33]. The following search permutation (see Textbox 1) was selected based on its ability to return a high number of relevant results.

Before conducting each search, Google settings were adjusted to deactivate results personalization based on prior activity, location, and stored data. Such adjustments reduce the probability of previous search activities by the researcher, or their location, affecting the results of the search [34]. Google alerts for once-a-month returns were also created for the keyword combinations to identify new results after the initial search period.

### Step 3: Screening

Based on preliminary searches, the 2 caregiving support databases, and particularly the 1 internet search engine, yielded a large number of results. In order to screen a feasible number of relevant results, we reviewed the first 100 results for each search, which accounts for the first 10 pages of results on Google with default settings [35,36]. In general, users interact most with first page results, with few visiting or clicking the following pages’ results [37]. Therefore, our approach goes beyond the typical use of internet search engines.

Duplicates were removed, and each returned result’s home page was previewed for eligibility. Search results that did not meet the eligibility criteria, such as information sheets that shared caregiving support information but not respite care contact information, news articles or general reports on respite care, were not included. When eligibility was unclear, the team discussed the service to determine if the result should be included.

### Step 4: Data Collection

Once screened, each eligible respite care organization’s website was saved and reviewed to extract information on the service eligibility criteria, service features, geographic availability, targeted demographics, costs, and language of the respite care service [5]. Similar variables have been identified and used in previous research [3,27,38,39]. To foster a consistent approach, the data collection was done independently by 1 researcher. If any discrepancies arose, issues were discussed and resolved with the research team.

### Step 5: Conducting Expert Consultations

Preliminary search strategy findings with a current list of services were sent to a group of 5 stakeholders (experts) comprising community members involved in respite care coordination and research. For review and feedback, experts were asked to verify our list of respite care services. They were also invited to direct us to any other respite care services in Quebec and identify any other essential feature required to describe the respite care services [39]. Stakeholders and identified organizations were also asked to provide feedback on the final paper and results.

### Step 6: Data Analyses

Qualitative deductive content analysis was used to descriptively analyze and interpret the data using a predetermined coding framework consisting of the following categories: service features, length of services, setting, care provider, region, costs, language, eligibility criteria, and user profile [40]. A geographical map of the services by region was created using graphic design software.

A framework defining “access” to health care was also identified post hoc as part of our iterative data analyses for further analyzing the data related to “accessibility” [41]. Norris and Aiken [41] conceptualized access to health care as characterized by (1) the family’s amenability to receive services (ie, the client’s readiness and knowledge of service and contextual factors), (2) the services’ availability (including location and hours of operation), (3) the eligibility of the client to access such services (including costs), and (4) the compatibility between the service and individual needs. This framework helped contextualize and structure our analysis of the findings, whereby each predetermined coding category was matched to 1 of the 4 components of health care accessibility.

## Results

### Overview

We used descriptions of the services along with specific service features, according to Norris and Aiken’s [41] framework of personal access to health care, to determine the overall accessibility of the respite care services identified—amenability, availability, eligibility, and compatibility.

### Amenability

**Figure 1:**
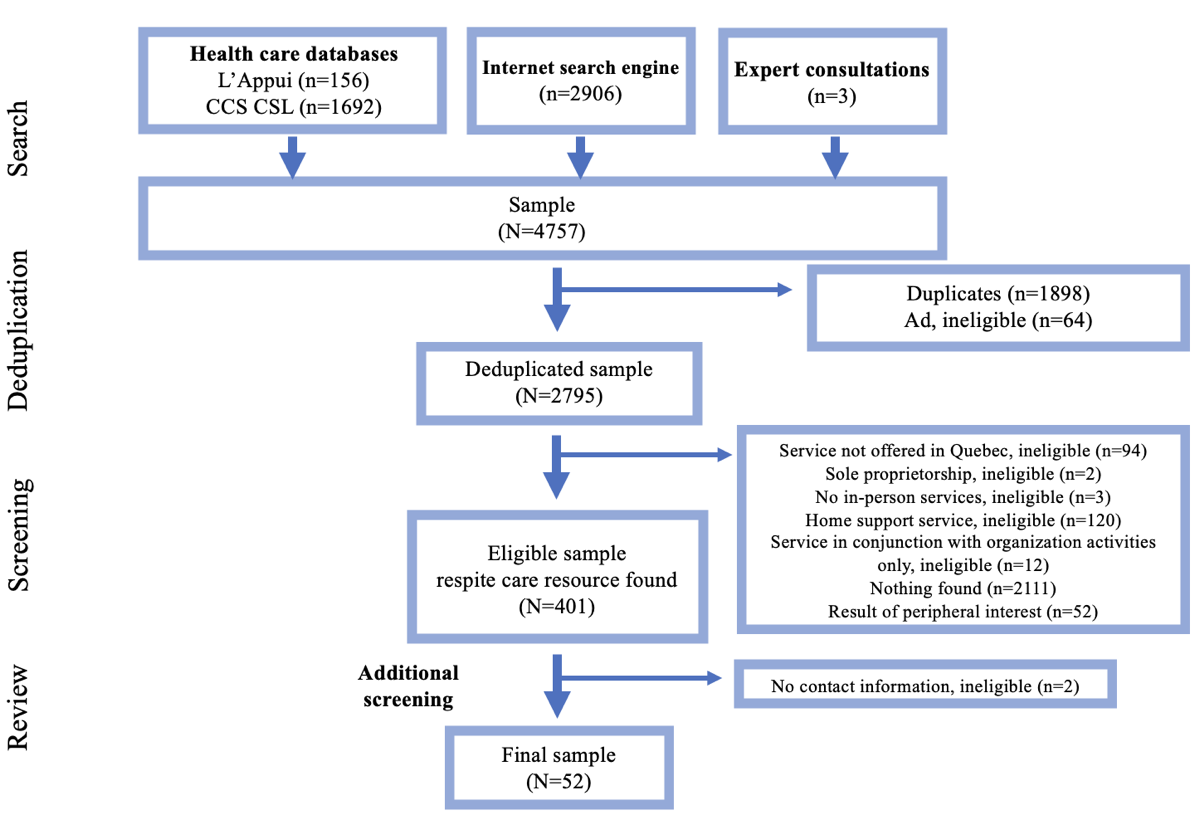
Flowchart of respite care data collection strategy, adapted from Moher et al’s model [42]. CCS CCL: Canadian Cancer Society Community Services Locator..

A total of 100 searches were conducted, including 41 on the Canadian Cancer Society Community Services Locator, 18 on the L’Appui Resource Directory, and 41 on Google (including monthly search alerts) producing a total of 4757 search results. Of these results, 401 results corresponded to respite care services, 52 of which were included in our analyses as they offered in-home respite care targeted to individuals with palliative care needs. The remainder of services were offered in a designated location (eg, a hospice or care home) and targeted to other key populations (eg, children with chronic disabilities). The most common reasons for exclusion were that respite care services were offered outside the province of Quebec (n=94), that home support services did not mention respite (n=120), and overwhelmingly, that no services specific to the concept of respite care were found on the website (n=2111). A total of 2 services were ultimately excluded due to providing no contact information. In some cases, these identified websites corresponded to an unrelated database, caregiving resource, news article, miscellaneous service, or obituary. Figure 1 summarizes the results obtained through data collection and screening.

Google was the most successful database for the identification of relevant respite care services. In fact, 40 eligible services (40/52, 76%) were discovered through Google, 26 of which (26/40, 65%) were exclusive to this search engine and were not found in the Canadian Cancer Society Community Services Locator or L’Appui databases. The Canadian Cancer Society Community Services Locator enabled the identification of 23 eligible services (8 exclusively), while the L’Appui Resource Directory identified 7 eligible services (1 exclusively). The expert consultations uncovered 3 services, 1 of which is currently in development, as well as others already identified through the other search strategies. An overview of each respite care service’s characteristics is explored in the following sections and summarized in Multimedia Appendix 1.

### Description of the Variety of Respite Care Services Offerings

The specific respite care offerings were characterized by a variety of activities and types of care for both the caregiver and the care recipient. All of these activities occurred in the context of a respite care visit, that is, this visit consisted of another person coming to the home to provide care for the care recipient so that the informal caregiver could leave the premises if they so desired.

The most common respite care activities offered were accompaniment (36/52, 69%), assistance with daily activities (29/52, 56%), personal care (19/52, 36%), and specialized care (17/52, 33%). In many cases, the organizations offered several types of specialized care, like palliative and cancer care. A total of 7 (14%) organizations specifically mentioned that they provided symptom and pain management as part of their respite care services. A total of 2 (4%) organizations listed restricted activities that they could not offer during the respite period (ie, medication administration and hygiene care). All organizations focused on assisting the care recipient. A total of 9 (17%) organizations also included some type of support for informal caregivers while on respite; for example, 1 organization had a rest lounge available for caregivers that the caregivers could visit while the respite care provider went to the care recipient’s home.

### Availability, Including Flexibility

**Figure 2:**
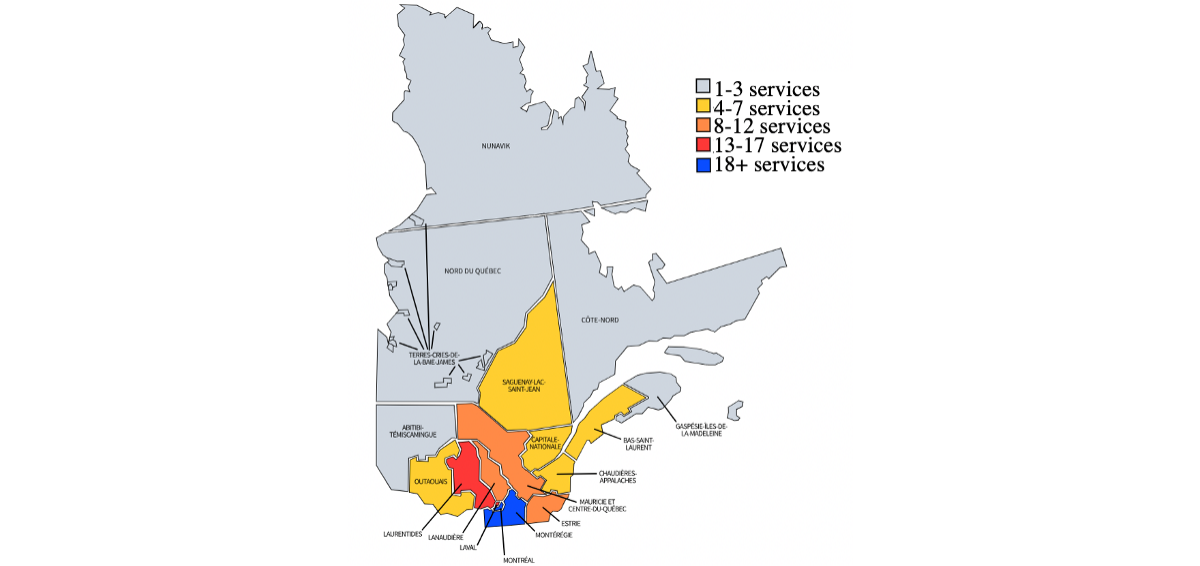
Locations of respite care services across Quebec health regions. The map was adapted from Qualifications Quebec and the Ministry of Health and Social Services [44,45]. Some services are offered in more than 1 region.

Respite care services were found primarily across Eastern Quebec, as can be observed in Figure 2. Only 1 service was identified in the regions of Abitibi-Témiscamingue, Nord-du-Québec, Nunavik and Terres-Cries-de-la-Baie-James. The greatest concentration of services was found in Greater Montreal, a densely populated metropolitan area comprised of the health regions of Montreal and Laval, as well as parts of Lanaudière, Laurentides, and Montérégie [43].

Agencies valued service flexibility. Many organizations emphasized individualized care for the unique situation of the family and explicitly specified that both planned and unplanned (emergency or on call) services were available (10/52, 19%). Some services had a designated telephone line for questions and service requests, available at all times (3/52, 6%). Furthermore, the majority of services report a 7-days-a-week (28/52, 54%), 24-hours-a-day (22/52, 42%) availability, for periods of a few hours (31/52, 60%), and at a frequency of once or twice a week (3/52, 6%). Some services explicitly advertised overnight respite services (9/52, 17%) and for lengths of over 24 hours (8/52, 15%). More flexibility with the number of hours and timing of the service was seen in some exceptional situations. A total of 4 (8%) organizations stated that overnight services were available for patients at the end of life. That said, it is unclear how quickly families can access respite care services identified in the sample due to delays between a request and service provision (14/52, 27%) due to requirements for initial consultations or waitlists.

### Eligibility

The care recipient target population for the identified respite care services consisted of persons in palliative care, at the end of life, persons with specific diagnoses like cancer, older adults, and persons affected by a loss of autonomy. Caregivers and loved ones were also targeted by the services, with some services citing specific eligibility requirements such as being a care recipient at the end-of-life, in palliative care, with a cancer diagnosis, or residing in a specific region. However, many organizations did not disclose their eligibility criteria (24/52, 46%).

A total of 20 (38%) respite care services were free of cost for the users, while 27 (52%) had associated fees. Often, these fees were not defined or openly available on the organization’s website (23/27, 85%), thus, requiring families to contact the respite care organization for more information. The disclosed fees ranged from CAD $15 (US $11.08) per day to CAD $32 (US $23.63) per hour, with the bottom range much lower than Quebec’s minimum hourly wage of CAD $15.25 (US $11.26) [46].

### Compatibility

Of the 52 services identified, 29 (56%) were offered by organizations classified as nonprofits as per Quebec’s Enterprise Register [47]. Respite care services often involved either volunteers (17/52, 33%); a team of multidisciplinary health professionals including patient care attendants and nurses (21/52, 40%); or a combination of volunteers and health care providers (4/52, 8%). Volunteer-provided services were most frequently free and accompaniment-based, whereas health care professional-provided services generally consisted of nursing-oriented care with associated fees. Nonetheless, staff, including volunteers, often had additional training for a specific clientele (23/52, 44%; eg, volunteers trained in end-of-life care). Agencies emphasized caregiver consistency and finding a good match between the caregiving dyad and staff.

A total of 29 (56%) organizations had a unilingual website (French or English) and 20 (38%) organizations had a bilingual website (French and English). The identified services’ websites often did not specify which languages were available for the provision of care (18/52, 35%), although some organizations specified language—some services were available only in 1 language (French, 9/52, 17%); others were bilingual (English and French, 17/52, 33%); or in 3 or more languages (8/52, 15%). Additionally, the websites of these services were not always available in the languages offered.

### Member-Checking Feedback From Organizations

An email was sent to respite organizations to confirm the findings of our research. A total of 15 (29%) organizations provided feedback on the results and validation for our project. Most frequently, organizations added additional information to what was provided on their website. For example, many organizations clarified the nature of activities performed during the respite period (5/15, 33%), the languages of services (6/15, 40%), or the availability and length of services (3/14, 21%). In some cases, the information gathered from the website was inaccurate and further clarified by the organization. For example, one organization provided fees that differed from that of their website.

## Discussion

### Overview

Respite care services should strive for high quality and safety. Norris and Aiken’s [41] framework of personal access to health care was identified post hoc and seemed to match our themes nicely. We analyzed the results based on how these services fit into this framework of accessibility. We will use the following section to discuss the gaps affecting amenability, variety of types of services, limited availability of services, and eligibility and provider impacts, as well as the limitations, strengths of our research, and opportunities for future research.

### Amenability: Gaps Affecting the Amenability of Respite Care Services

The need for advanced digital health literacy skills, incomplete information and language barriers are some of the key gaps affecting the amenability of respite care services that were identified as part of this environmental scan.

A significant time investment and high digital literacy skills were required to carefully create search strategies, sift through thousands of results, and retrieve a relatively small selection of respite care services relevant to palliative care families in Quebec. Even a sophisticated user may not have the mindset required to go through a tedious search process given the demanding nature of caregiving in the palliative care context. Caregivers or clinicians may not be familiar with internet search strategies or be in a position to dedicate time and energy to the search and identification process for respite care services. Thus, the intensive search and screening process constitutes an important accessibility barrier, needing to be addressed as part of best practice guidelines, and perhaps alleviated with the use of collaboratively developed digital tools (eg, chatbot) or care navigators [7,19,48,49].

Incomplete access to information was one of the most significant challenges encountered in this project. Many of the identified respite care organizations’ websites did not share critical information on their services, such as the fees, eligibility criteria, or availability. In some cases, this missing information could be obtained by reaching out to the organization directly. However, many organizations did not respond to our request for feedback. Incomplete information on respite care services is a deterrent to access, often resulting in caregivers having unanswered questions and unclear expectations [50]. In other words, caregivers may find it difficult to gauge if the respite care service is relevant to their unique situation, if they are eligible and what procedure they should follow to access the service. Clinicians may also find it difficult to know what services are available in the community, where to link or refer their clients and what the request procedure looks like. This challenge may be accentuated when respite care organizations’ websites are only available in 1 language, as was seen in our sample. With this in mind, respite care organizations may wish to make information about their services more widely available online and continue to incorporate clinician, caregiver, and care recipient feedback, so that their services can become more widely accessible, available, and family-centered [2,48].

### Types of Services: Complex Variety of Respite Care Service Offerings

Respite care services take on many formats and should aim to address a variety of individualized needs [5,6,48,51,52]. Frequently cited priorities for end-of-life care at home include physical (eg, symptom management) and psychosocial care (eg, interpersonal connection), which nurses are often best equipped to provide [2,6,16,50,51,53,54]. Service offerings aimed at caregivers, like rest lounges or psychological care, may help to alleviate the caregiver burden in targeted ways so that they can be better equipped to cope and care for their loved one [9,52].

Our sample of 52 respite care services offers an array of respite care services and reflects the various priorities of families in a palliative stage of care. This data contradict the common criticism of respite care, that it is too often focused solely on caregiver needs and burdens while ignoring the care recipient’s needs [2]. These findings also suggest that services have the potential to address a wide range of needs and provide caregivers with greater flexibility to choose how they want to spend their time while on respite [3]. However, the variety in respite care service descriptions may make it difficult for users and clinicians to compare and contrast options in their community, and perhaps select what they need. Systematic reporting of basic services across all service providers, such as an easy-to-search database that is regularly updated, is needed to determine the best types of service provision. Organizations should also troubleshoot how to deliver effective services within a low-cost model, in an effort to improve at-home respite care across Quebec regions.

### Availability: Limited and Sparse Availability of Services

Rural and Indigenous communities are often faced with service provisions not meeting the needs and preferences of families, nor supporting death at home [20,52]. Our research identified a lack of in-person services in Western and Northern Quebec, areas representing approximately 2.2% of Québec’s population (estimated population of 195,409 in 2022) and 2.5% of Quebec’s deaths every year (1719 deaths in 2021) [43,55]. These findings accentuate the scarcity of resources described in the literature [56]. Therefore, to enable more caregiving dyads to benefit from adapted respite care, infrastructure, targeted funding, and service options need to be expanded, particularly in rural and Indigenous communities of Quebec. Inclusivity, community leadership, and family-centered approaches should take the forefront in these efforts [56].

Best practice guidelines for the provision of respite care stipulate that flexibility in service provision is a key criterion to ensure that the ever-changing needs of the caregiver and care recipient are being met, that a continuum of care is maintained, that the diversity of the clientele is respected and that caregiving dyads can make the most of the respite period [5,6,19,48]. In Quebec, the length of the 52 identified services’ availability and frequency were diverse. Flexible services, such as those available 24 hours a day and 7 days a week, for a longer period of time (hours to days) or with “on-call” availability, may enable the caregiving dyad to engage in a greater selection of activities (eg, sleeping, running errands, and social interactions), as opposed to respite care services limited to a specific time of the day. The services’ flexibility also potentially impacts how quickly caregivers can access respite care when an urgent or unplanned need arises, to ensure the care recipient is still being cared for. Given the results’ significant range of availabilities, we can conclude that some services are as flexible as current guidelines suggest. These results point to a larger issue of gaps and barriers affecting the accessibility and usage of respite care services.

### Eligibility: Eligibility Requirements Limiting Access to Respite Care Services

The respite care clientele is diverse [11]. Hence, eligibility requirements have the potential to restrict access to families most likely to use such home-based nursing services, such as users with less financial resources or those who speak minority languages [3,7,16,20]. Services without specific eligibility criteria or free of charge may be more appealing to a greater population of families in need of respite. The organizations specifically mentioning eligibility criteria may help to ensure the population of a given region has access to services in their community, or that the respite care services meet the needs of that specific population (ie, services tailored to people at the end-of-life). However, the respite care services that were most flexible, were also generally fee-based. Consequently, financial barriers may limit access to such services for families who need them most.

### Compatibility: Provider Impact on the Compatibility of Respite Care Services

The therapeutic relationship between the caregiver, care recipient, and respite care provider is essential in achieving satisfaction with a respite care service and is tightly linked with caregiver well-being [5,7,8,19,57]. Several organizations highlighted caregiver consistency in their description of services, which may play an important role in the development of a collaborative and trusting relationship. Thus, these results may prompt organization leaders to consider diversifying multidisciplinary teams, provide further training and aim for greater care provider consistency in an effort to achieve high-quality respite care service provision.

As suggested within best practice guidelines for respite care, service providers must be equipped with the skills, training, and experience to provide safe and high-quality care [5,19,48,57]. Volunteers are great resources for respite care service provision but may be limited in the offerings they are allowed to provide [8,48,58]. This may explain why many identified volunteer-provided respite care services in our sample were oriented around accompaniment, a service that may be more personalized and adaptable to a client’s unique psychosocial needs [59]. Volunteer limitations may also provide context for the restrictions in service provision, a potential deterrent for families, depending on their care requirements [5]. On the other hand, health care providers have the benefit of training and experience with structured, specialized interventions corresponding to the common requests of caregivers, the needs of the care recipient, and the specialized nature of palliative care in general [50,51,54,59]. Nurses, in particular, are heavily involved in clinical, coordination, and leadership positions associated with palliative care and home care services due to the holistic nature of their role and therapeutic relationship with families [60,61]. In our sample of services, health care professional-provided services were often centered on physical care, symptom management, and other nursing interventions. Collaborations between health care professionals and volunteers, as seen in the sample, potentially contribute to providing cost-effective and family-centered respite care, while overcoming challenges associated with limited health care resources [60]. Similarly, additional training, showcased by some agencies, may further clarify the roles and responsibilities of the care provider while enabling them to provide high-quality care specifically targeted to individuals with cancer, at the end-of-life or in palliative care, for example [48]. This centralized information about respite care providers may encourage involvement in local respite care organization activities, for example, by creating a network of respite care providers and collaborative training opportunities.

### Limitations and Strengths

Limitations to this research include the availability of information on the internet, the use of specific languages (ie, only English and French), limited data collection (ie, restricting to 3 search databases or engines, and 100 results per return), and the impact of digital algorithms. This analysis represents only the information available on the internet and feedback from a limited number of organizations, which may slightly differ from actual respite care service features or currently available services. Many excluded services highlighted the provision of home care services without specifically mentioning a respite component. Therefore, relevant services that provide respite care without explicitly advertising these services may have been excluded. However, “respite care” is the term most commonly found in the literature and that caregivers are most likely to use when seeking a break from their dyadic informal caregiving roles [2,8]. Future research could examine subsets of this project’s ineligible services (ie, home care services and respite care services outside the home) in an effort to better understand the breadth of community health care services available to informal caregivers and care recipients.

Additionally, due to resource constraints, not every postal code could be searched in the L’Appui Resource Directory, and only the first 100 results for each search were screened. This cut-off point was chosen in other grey literature searches and justified by the amount of traffic received by results on the first page of Google compared to any additional pages [35,36,62]. Nonetheless, there is still the possibility that some less popular or poorly advertised respite care services may have been missed in the search process. Similarly, Google algorithms may have played a role in the display and order of search results despite taking precautions to disable such alterations. However, we believe our multimethod approach helps to overcome these limitations.

All things considered, the environmental scan methodology devised for this project was successful at identifying diverse at-home palliative respite care services across the province of Quebec and synthesizing service features [21,63]. Novel methodologies used in the project, such as combining search engines and internet-based community health databases, using postal codes to search for services, as well as seeking expert feedback via member-checking, may be useful for other researchers attempting to comprehensively map other types of services while reducing bias [64]. There is also a potential to further expand our search strategy by including other tools (eg, Google Maps and artificial intelligence chatbots), strategies we have attempted but ultimately abandoned due to the lack of existing methodologies and the current functioning of these tools not showcasing relevant results.

### Opportunities for Future Research

Caregivers are often challenged by overwhelming amounts of information when seeking health care services on the internet, hence, a coordinated database is an important unmet caregiving need [6,7,19,53]. Therefore, concise and complete records of respite care services are warranted to (1) improve families’ knowledge of the services available in their community and how to access them, (2) to improve clinicians’ ability to share and refer clients to such services, and (3) to promote the expansion of existing services and development of complementary resources [49]. Best practice guidelines and digital databases should be updated, further developed, and validated by users and organizations, to reflect health care service search and identification challenges. For example, key filters like type of respite care provider, fees, service offerings, and eligibility criteria, could be included as part of a digital database. Moreover, the methodology and findings may be of interest to referring clinicians and policymakers responsible for planning future needs as Canada moves away from institutional care, toward holistic community care.

### Conclusions

Comprehensively identifying available respite care services is essential for assessing the overall availability of respite services, as well as identifying potential barriers that individuals and clinicians face when seeking out these services [4,48]. The findings of this project emphasize that the identification, navigation, and access to such services likely remain challenging for individuals in need of respite and clinicians looking to refer their patients. These results stress the need for a centralized searchable database to render accessible information on respite care services available in communities across Québec. The proposed methodology, consisting of combining several data sources, may guide researchers in conducting other community health service environmental scans.

## Appendix

Multimedia Appendix 1 List of 52 Quebec respite care services offered at home to families with palliative care needs identified via Google, in the Canadian Cancer Society Community Services Locator or the L’Appui Resource Directory.
