# Supplementary material for: In-Home Respite Care Services Available to Families With Palliative Care Needs in Quebec: Novel Digital Environmental Scan
Source: JMIR Nurs. 2024 Apr 16;7:e53078. doi: 10.2196/53078 (PMC11061788; doi:10.2196/53078)
Supplement: Multimedia Appendix 1 [file nursing_v7i1e53078_app1.docx]

**Multimedia Appendix 1.** List of 52 Quebec respite care services offered at home to families with palliative care needs identified via Google, in the Canadian Cancer Society Community Services Locator or the L’Appui Resource Directory.

Please note that an updated directory can be found at <https://doi.org/10.5683/SP3/HOAKSC>

| **Name of Organization** | **Type of Organization** | **Setting** | **Service Features** | **Length of Services** | **Care Provider** | **Region** | **Costs to User** | **Language** | **Eligibility Criteria** | **User Profile** | **Source** |
| --- | --- | --- | --- | --- | --- | --- | --- | --- | --- | --- | --- |
| Accès Services Santé | Business Corporation or Company | In-Home CHSLD Hospital Private Residences | Respite care including accompaniment to appointments, housework, hygiene care, assistance with medical treatments, supervision, companionship & specialized care (i.e. palliative care); Planned and unplanned services available | Minimal length of 1hr for nursing care, 3hrs for assistance services | Nurses, LPNs, PABs, family & social | Longueuil Québec Montreal Montérégie Basse Laurentides | Fees (not specified) | Website available in FR & EN | Not specified | Individuals weakened by age, loss of autonomy, illness or disability | Google |
| Accueil Sérénité | Non-Profit Organization | In-Home | Respite care in the form of palliative and end-of-life accompaniment | 3hrs/week during the day in the palliative stage; More flexibility (number of hours, time of day, day/evening/night) at the end of life | Volunteers trained in at-home accompaniment | Bellechasse | Not specified | Website only available in FR Service only available in FR | Not specified | Individuals with a cancer diagnosis, bereaved, in palliative care, at the end of life & their loved ones, caregivers | CCS |
| Affinity Health | Not Specified | In-Home | Respite care including personal care, companion services, grocery shopping, outings, coordination of medical supplies/equipment; Associated palliative care including pain relief, health monitoring, grief counseling, meal preperation, etc. | No minimum hours 24/7 on-call nursing support | PABs, HSWs, companions, nurses & housekeepers | Greater Montreal Gatineau/Hull | Fees (29$/hour) | Website only available in EN | Not specified | Elderly individuals | Google |
| Agence SAO | Business Corporation or Company | In-Home CHSLD Hospital Private Residences | Respite & palliative/end-of-life care | 24hrs/day, 7 days/week availability | Professionals (PAB, family workers, social workers) with additional training | Trois-Rivières | Not specified | Website only available in FR | Not specified | Individuals wanting to stay at home despite loss of autonomy & with cognitive disorders, Alzheimer's or Parkinson's | Google |
| Albatros Capitale-Nationale | Non-Profit Organization | In-Home CHSLD Hospital Private Residences | Respite care in the form of activities & exchanges depending on person's abilities, as well as ccompaniment of caregiver before/after loved one's death | Few hours | Volunteers trained in palliative care | Quebec Portneuf Côte-de-Beaupré | Free | Service available FR, EN | Person must be aware of their palliative care diagnosis Person and loved ones must wish to benefit from services Person must have a palliative diagnosis | Individuals at the end-of-life, in palliative care | Google |
| Albatros Drummondville | Non-Profit Organization | In-Home CHSLD Private Residences | Respite care can be provided through palliative pet therapy; Do not offer hygiene care & can only administer medication that has been pre-prepared by the caregiver | Few hours | Volunteers trained in palliative care | Drummondville Centre-du-Québec | Free | Fees (29$/hou+H7) | Not specified | Individuals at the end-of-life, in palliative care & their loved ones, caregivers | Google |
| Albatros en Montérégie | Non-Profit Organization | In-Home CHSLD Hospital Private Residences Palliative Care Homes | Not specified | Once per week | Volunteers trained in palliative care | Montérégie Longueuil | Free | Website only available in FR | Carrier of serious illness or at the end of life Wish to be accompnied by the patient and/or their family | Individuals with a serious illness, at the end-of-life, in palliative care & their loved ones, caregivers | Google |
| Albatros Lévis | Non-Profit Organization | In-Home CHSLD Hospital | Respite care in the form of accompaniment, help with daily tasks/activities according to the person's health status & energy levels | Half-day/week | Volunteers trained in palliative care | Grand Lévis Berthier-sur-Mer Saint-Raphaël Sainte-Claire Sainte-Hénédine Saint-Lambert-de-Lauzon Saint-Apollinaire Saint-Anselme | Free | Website only available in FR Service available in FR, EN, Spanish | All persons critically ill or at the end of life | Individuals in palliative care & their loved ones, caregivers | CCS |
| Albatros Mont-Laurier Albatros Vallée-de-la-Gatineau | Non-Profit Organization | In-Home Outside the home Facility | Not specified | Less than 24hrs (3-4 hrs), day & evening, 7 days/week availability | Not specified | Montcerf-Lytton Mont-Laurier  Laurentides | Free | Website only available in FR Service only available in FR | All persons at the end of life, in palliative care | Individuals with a serious illness, at the end-of-life, in palliative care & their loved ones, caregivers | L'Appui |
| Albatros Montréal | Non-Profit Organization | In-Home CHSLD Hospital Private Residences Palliative Care Homes | Respite care with spiritual approach & attentive listening | Half-day/week (fixed or variable), day & evening, 7 days/week availability | Volunteers trained in palliative care | Montreal | Free | Website only available in FR Service available in FR, EN | All persons critically ill, in palliative care or at the end of life | Individuals with a serious illness, at the end-of-life, in palliative care & their loved ones, caregivers | Google L'Appui |
| Albatros Trois-Rivières | Non-Profit Organization | In-Home CHSLD Hospital Private Residences Palliative Care Homes | Respite care in the form of a attentive and comforting presence | Day & evening, 7 days/week (night exceptionally) availability; Accompaniment 3-4hrs, once per week, | Volunteers trained in palliative care | Trois-Rivières | Free | Website only available in FR Service only available in FR | Care recipient must be critically ill, in palliative care or at the end of life | Individuals with a serious illness & their loved ones, caregivers | Google |
| Bien Chez Soi | Sole Proprietorship | In-Home Outside the Home | Respite care in the form of medical interventions, housework, hygiene care, palliative accompaniment, support & nanny service for children | 24hrs/day, 7 days/week availability; Schedule planned 1 month in advance | Patient care attendants, nannies, family support attendants, nurses & cleaning attendants trained in palliative care | Bas-Saint-Laurent Capitale-Nationale Chaudière-Appalaches Côte-NordEstrie Launaudière Laurentides Laval Mauricie Montérégie Montreal Outaouais Saguenay | Fees (not specified) | Service available in FR, EN Request for other languages can be made | According to individual needs | Various | CCS |
| Big Hearts Homecare | Not specified | In-Home | Respite care including assistance with bathing, dressing, feeding, toileting, meal preperation, medication reminders, companionship, housekeeping, transportation, pain/medication/symptom management, breathing techniques, healing touch, visualization, nursing coordination/consultation; Planned and unplanned services available | Few hours to months; Phone line and services available 24 hrs/day, 7 days/week | Qualified & experienced caregiver | Montreal | Fees (not specified) | Website only available in EN Request for specific language can be made | Not specified | Various (elderly, frail, housebound, terminally/chronically/acutely ill, physically/mentally challenged, individuals recently discharged from hospital, family caregivers, individuals requiring assistance caring for their newborn/children, developing life skills, home maintenance & accident victims) | Google |
| Groupe Santé Carole Paquette | Business Corporation or Company | In-Home CHSLD Hospital Private Residences | Respite care in the form of assistance with daily tasks, surveillance, errands, accompaniment, hygiene care, meal preparation, medication reminders, housekeeping & mobilization assistance; Palliative care offered as part of nursing care (includes MAID) | Few hours (minimal length of 3 hrs) to 24 hr/day care; Phone line and service available 24 hrs/day, 7 days/week | PABs & nurses | Brossard | Fees (not specified) | Website available in FR, EN Service available in FR, EN | Not specified | Various | Google |
| Centre d'Action Bénévole de Farnham | Non-Profit Organization | In-Home At Organization | Respite in the form of palliative accompaniment | Less than 24hrs; Day availability only (Monday to Friday) | Volunteers trained in palliative care | Montérégie Estrie Farnham Ange-Gardien Saint-Brigide-d'Iberville Sainte-Sabine | Free | Website only available in FR Service available in FR, EN | Reserved to members | Various | L'Appui CCS |
| Centre d'Action Bénévole Le Hauban | Non-Profit Organization | In-Home Outside the Home Hospital | Respite in the form of home assistance, psychosocial support, end-of-life accompaniment & workshops for caregivers | Blocks of 3-4hrs; Day, evening, night availability; 7 days/week | Volunteers trained in palliative care | Gaspé Gaspésie-Îles-de-la-Madeleine | Free | Website only available in FR Service available in FR, EN | Priority given to individuals over 65 Must be in palliative care, in the last 2 months (or less) of life, must provide references from the healthcare team | Various | Google L'Appui |
| Code Violet | Business Corporation or Company | In-Home Outside the Home Facility | Respite care, palliative care & provision of nursing care including perfusions, dressings, pressure monitoring, ostomy care, hygiene care, meal preparation, assistance with daily tasks, mobilization, social activities, light housekeeping, accompaniment to improve autonomy & comfort; Planned and unplanned services available | Few hours (minimum length of 3hrs) to several days; Phone line available 24 hrs/day, 7 days/week | Nurses, LPNs, PABs, home support workers, physiotherapists, occupational therapists, housekeepers & food aides | Montreal  Quebec  Drummondville | Fees (not specified) | Website available in FR, EN | None | Various | Google |
| Comité d'Accompagnement La Source | Non-Profit Organization | In-Home Hospital | Respite in the form of positive & reassuring presence | 7 days/week availability | Volunteers | MRC Rivière-du-Loup | Free | Website only available in FR | Individual with advanced cancer diagnosis, in palliative care and loved ones | Individuals at the end of life, with an incurable disease & their loved ones | CCS |
| Coopérative de Services à Domicile de l'Estrie | Cooperative | In-Home | Respite in the form of presence & supervision at the end-of-life (staying at the bedside), accompaniment to activities & stimulating activities | Day, evening & night, 7 days/week availability | PABs | Sherbrooke  Fleurimont Bromptonville St-Élie Rock-Forest Deauville Lennoxville Des Nations | Fees (not specified) | Website only available in FR | Priority to individuals 65+, with loss of autonomy (temporary or permanent) or with physical disability (temporary or permanent) | Older adults and individuals with loss of autonomy (permanent or temporary) | Google |
| Douceur de Vivre Soins à Domicile | Sole Proprietorship | In-Home | Respite in the form of palliative care | 7 days/week, day & evening availability | Not specified | Montreal South Shore Saint-Catherine | Not specified | Website only available in FR | Not specified | Not specified | Google |
| Elite Comfort | Business Corporation or Company | In-Home Hospital Retirement Home Convalescent Home | Respite in the form of home care, emotional, moral support, housekeeping, personal care & companionship | 24 hrs/day, 7 days/week availability; Minimal length of 2 hrs; On-call in case of emergency | Caregivers, companions, attendants, nursing assistants & nurses | West Island Downtown Montreal & East Laval & North Shore Montérégie Quebec City | Fees (not specified) | Website available in FR, EN | None | Inndividuals recovering from illness/operation, limited mobility, disability, individuals with dementia, at the end-of-life | Google |
| Équipe d'Accompagnement au Diapason | Non-Profit Organization | In-Home | Respite care in the form of a visit for support, listening & accompaniment | 3-4 hours per week | Volunteers | Cowansville MRC Brome-Missisquoi Haute-Yamaska territory | Free | Website only available in FR Service available in FR, EN | None | Individuals at the end-of-life & their loved ones | CCS |
| Gold Squad | Business Corporation or Company | In-Home | Respite care in the form of in-home services, including palliative care | Not specified | PABs, caregivers & nurses trained in PDSB, first aid & with experience with dementia, Parkinson's, neurological disorders | Greater Montreal | Fees (not specified) | Website available in FR, EN | Not specified | Older adults | Google |
| Golden Home Care | Business Corporation or Company | In-Home Assisted Living Facilities Hospital | Respite in the form of home care support (companionship, meal preparation, light housekeeping, managing home administration & pet care) | 24hrs/day, 7 days/week availability; Services available on short notice; Hourly, full-time, short or long-term basis (overnight also available) | Caregiver trained in first aid, CPR, PDSB, SPVM | Dorval | Fees (not specified) | Website available in FR, EN | Not specified | Seniors & family, individuals suffering from an illness/disease, recovering from surgery, individuals experiencing challenges | Google |
| Graceful Living Home Care Services | Sole Proprietorship | In-Home | Respite care in the form of personal care services, meal preparation, medication reminders, light housekeeping, accompanied outings & specialized services (e.g. palliative care); Palliative care includes comfort care, positioning, companionship, meal preparation, personal hygiene , security & communication with family | 24hrs/day, 7 days/week availability; Hourly care, part or full time care available up to 24 hr care | Caregivers | Montreal West Island East End Laval | Fees (not specified) | Website only available in EN | Not specified | Older adults | Google |
| Home Care Assistance (Soins à Domicile Montréal) | Business Corporation or Company | In-Home | Palliative respite care in the form of comfort care, mobilization, companionship, meal preparation & personal hygiene | 24hrs/day, 7 days/week availability | Nurse's aides & retired nurses | Westmount Mount-Royal West Island Laval South Shore | Fees (not specified) | Website available in FR, EN | Not specified | Older adults | Google |
| Homecare Solutions Soins à Domicile | Sole Proprietorship | In-Home | Respite in the form of personal support, assistance with home maintenance & tailored home care plans; Palliative care including respite, nursing assessment, management of pain/symptoms, client support, advocacy & clinical skills | On-call services 24/7; 24hrs/day, 7 days/week availability | Physician & nurses | Greater Montreal | Fees (not specified) | Website available in FR & EN Service available in FR, EN, Italian | Not specified | Seniors & family | Google |
| In Home Care Group | Business Corporation or Company | In-Home | Respite care in the form of home help (cooking, housework, laundry), personal care (hygiene, oral care, toileting) & nursing care (medication administration, dressing changes, ventilator care, pain & disease management) | Few hours to 24 hrs/day | Not specified | Montreal South Shore North Shore Laval | Fees (not specified) | Website available in FR, EN Service available in FR, EN | Not specified | Individuals with a loss of autonomy | Google CCS |
| La Rose des Vents | Non-Profit Organization | In-Home | Respite care in the form of assistance with daily activities (errands, bathing, laundry, meal preparation) | 1 meeting of 4 hrs/1-2 weeks | Social/domestic aids & volunteers | Sherbrooke MRC Sherbrooke Estrie | Fees (not specified) | Website only available in FR Service available in FR, EN | Anyone living with cancer or living with someone with cancer (loved one, caregiver) Anyone experiencing bereavement related to cancer | Individuals who have cancer, caregivers who need help with daily tasks/activities | Google L'Appui CCS |
| Ligne de Vie du Témiscouata Inc. | Non-Profit Organization | In-Home CHSLD | Respite & palliative care in the form of accompaniment & visits | Not specified | Not specified | Témiscouata-sur-le-Lac MRC Témiscouata | Not specified | Website only available in FR | Not specified | Individuals in the pre-terminal & terminal phases of illness | CCS |
| Maison Soutien aux Aidants | Non-Profit Organization | In-Home Nursing Home Hospital Day Centre | Respite in the form of assistance with daily living activities, social life, securing presence & accompaniment; Group respite, group activities and day center also available | 24 hrs/day, 7 days/week availability | Trained respite companion (volunteers + employees) | Haute-Yamaska La Pommeraie | Fees (not specified) | Website only available in FR Service available in FR, EN | Not specified | Caregiver of individual with illness, older adult, or a loss of autonomy | CCS Google |
| Multi-Aide Estrie | Business Corporation or Company | In-Home | Respite including end-of-life care | 24 hrs/day, 7 days/week availability; Short, medium or long-term duration | Social workers, specialized educator & PABs | Magog  Estrie | Fees (not specified) | Website only available in FR Service only available in FR | Individuals with cognitive and/or physical losses | Individuals with cognitive and/or physical losses, older adults | Google |
| NOVA l'Ouest de l'Île | Non-Profit Organization | In-Home | Respite in the form of mobilization, hygiene care, laundry & meal preparation; Respite also available when caregivers participate in organization activities and at a day center | 24 hrs/day, 7 days/week availability; Minimum of 3 consecutive hours | Home health aide | West Island Baie-d'Urfé Beaconsfield Dollard-des-Ormeaux Dorval Île-Bizard Île-Perrot Kirkland Pierrefonds Pincourt Pointe-Claire Roxboro Saint-Anne-de-Bellevue Saint-Geneviève Senneville Lachine | Fees (14$/hour) | Website available in FR, EN Service available in FR, EN | Not specified | Vulnerable adults, youth & community | CCS |
| Nova Soins à Domicile | Non-Profit Organization | In-Home | Respite in the form of assistance with daily activities, personal hygiene, mobility, domestic help, accompaniment, social support for caregivers, some treatments & medication administration; Restrictions related to household cleaning, preparation of complex meals, administering injections, changing sterile dressings, giving enemas, administering oral medication & providing foot care | 24hrs/day, 7 days/week availability | Nurses, home care attendants, social workers & volunteers | Montreal | Free | Website available in FR, EN Service available in FR, EN | None | Individuals ill or at the end or life, with cancer with a loss of autonomy | Google CCS |
| Palli-Aide-Accompagnement en Soins Palliatifs du Saguenay | Non-Profit Organization | In-Home Private Residence CHSLD Palliative Care Home Hospital | Respite in the form of palliative accompaniment, active listening & support; Mobile day center offering massage therapy, hairdressing services & creative workshops | 3 hrs/week | Volunteers trained in palliative care | Chicoutimi | Free | Website only available in FR Service only available in FR | Patient aware of their condition and willing to receive support from a volunteer | Individuals in palliative-stage cancer or at the end-of-life (any illness) & loved ones/caregivers | CCS |
| Pallia-Vie | Non-Profit Organization | In-Home | Respite care consisting of assistance with daily tasks for either patient or caregiver | Not specified | Volunteers | Saint-Jérôme  Pays-d'en-Haut Rivière-du-Nord Thérèse-de-Blainville Argenteuil Mirabel | Free | Website only available in FR | Patient must have been diagnosed with cancer or a degenerative disease with a terminal outcome Must be living in the regions highlighted in the "region" column | Individuals with cancer, degenerative diseases, caregivers, bereaved | Google |
| Palliacco Mont-Tremblant | Non-Profit Organization | In-Home | Respite in the form of accompaniment, support & comfort; Caregivers can rest at the organization's lounge | Day & evening availability; Night availability at the end of life; Blocks of 3-5hrs (or 8hrs at the end of life) | Volunteers trained in palliative care | Mont-Tremblant MRC des Laurentides MRC des Pays-d'en-Haut MRC Antoine-Labelle | Free | Website available in FR, EN Service available in FR, EN | Caregivers of individual in palliative care or with cancer | Individuals with cancer, at the end of life & caregivers | CCS L'Appui |
| Palliacco Sainte-Agathe-des-Monts | Non-Profit Organization | In-Home | Respite in the form of accompaniment, support & comfort; Caregivers can rest at the organization's lounge | Day & evening availability; Night availability at the end-of-life; Blocks of 3-5hrs | Volunteers trained in palliative care | Ste-Agathe  MRC des Laurentides MRC des Pays-d'en-Haut MRC Antoine-Labelle | Free | Website available in FR, EN Service available in FR, EN | Not specified | Individuals with cancer, at the end of life & caregivers | Google CCS |
| Palliacco Saint-Sauveur | Non-Profit Organization | In-Home | Respite in the form of accompaniment, support & comfort; Caregivers can rest at the organization's lounge | Day & evening availability; Night availability at the end-of-life; Blocks of 3-5hrs | Volunteers trained in palliative care | St-Sauveur  MRC des Laurentides MRC des Pays-d'en-Haut MRC Antoine-Labelle | Free | Website available in FR, EN Service available in FR, EN | Not specified | Individuals with cancer, at the end of life & caregivers | Google CCS |
| Présence Lotbinière | Non-Profit Organization | In-Home CHSLD Private Residences | Respite in the form of palliative accompaniment & support | 24hrs/day, 7 days/week availability | Volunteers trained in accompaniment with additional PDSB, CPR training & experience with physical care, comfort care & cognitive diseases | St-Flavien | Free | Website only available in FR Service only available in FR | Individuals with a cancer (diagnosis - up to 5 years after remission) | Individuals with a loss of autonomy (physical/cognitive), incurable disease, cancer in palliative care, at the end-of-life & loved ones | Google CCS |
| ProJ Soins | Business Corporation or Company | In-Home | Respite care in the form of accompaniment & supervision | 24 hrs/day, 7 days/week availability; On-call 24 hrs/day, 7 days/week; Minimal length of 1hr | Family auxiliaries, health/social services auxiliaries, PABs & nursing assistants trained in PDSB & CPR | Montreal | Fees (not specified) | Website only available in FR | Not specified | Autonomous older adults, individuals in loss of autonomy who wish to remain in their home, individuals having recently undergone surgery, are ill, at the end-of-life, with degenerative diseases & caregivers who need a break | Google |
| Répit-Ressource | Non-Profit Organization | In-Home | Respite in the form of presence, supervision at the end of life (staying at bedside), companionship & stimulation (via games, discussions, activities) | Day & evening availability | Trained attendants | Montreal East Rosemont Hochelaga Maisonneuve Anjou Mercier Est + Ouest Pointe-aux-Trembles Rivière des Prairies | Fees (not specified) | Website available in FR, EN Service available in FR, EN | Not specified | Older adults, people with disabilities, families & young professionals | Google CCS |
| Retraite à Domicile | Business Corporation or Company | In-Home Hospital Residence | Respite care in the form of personal care services, housekeeping, supervision, shopping | On-call services 24 hrs/ day, 7 days/week; Day, evening, night & weekend availability | Nurses, nursing assistants, PABs, companions & housekeepers | Montreal Laval South Shore | Fees (26$/hour) | Website available in FR, EN Service available in FR, EN | Not specified | Older adults | Google CCS |
| SE Health - Elizz | Non-Profit Organization | In-Home | Respite in the form of personal care (bathing, feeding, toileting, dressing, management of medication, assistance with daily activities), cognitive stimulation; Specialized in cancer & palliative care | Minimal length of 3hrs; 24hrs/day, 7days/week availability | Nurses, nursing assistants, PABs & companions | Montreal | Fees (not specified) | Website available in FR, EN Service available in FR, EN, other | None | Older adults | CCS Google L'Appui |
| Organismes René-Verrier | Non-Profit Organization | In-Home | Respite in the form of palliative accompaniment, support, active listening, activities, supervision, meal preparation, personal care, nursing care, hygiene care & medication administration; No housework | 24hrs/day, 7days/week availability | Nurses, nursing assistants, PABs & companions | Drummondville | Free | Website only available in FR Service only available in FR | At the end of life Care recipient must have a degenerative disease with terminal outcome Must reside in the area of Drummondville | Individuals at the end of life & their loved ones | Google CCS |
| Palliative Home-Care Society of Greater Montreal | Non-Profit Organization | In-Home | Respite in the form of assistance with daily activities, a comforting presence, psychological care for caregivers; Includes pain/symptom management, follow-up, comfort care, hygiene care | 24 hrs/day, 7 days/week availability; Maximum of 6 hrs/week | Nurses aid & trained volunteers | Montreal | Free | Website available in FR, EN Service available in FR, EN, Spanish, Créole, Italian, other (according to availability) | Individuals living with a pre-terminal/terminal cancer Residing on the Island of Montreal, Laval or South Shore Request made by the CLSC (preferred, required for Laval/South Shore) or directly Respite reserved for patients admitted to the society for palliative and end-of-life care | Individuals with cancer or other illness in advanced stage & caregiver | Google L'Appui CCS |
| Soins à Domicile Personnalisés | Business Corporation or Company | In-Home Hospital Nursing Homes Assist-Living Facilities Community/Social Service Organizations | Respite care in the form of companion care, medication reminders, light cleaning, shopping, cooking & hygiene care; Palliative assistance (one of their expertise) involves coordination of medical and non-medical services | Hourly, shorts stays (3 hrs), overnight or live-in; Services on call 24hrs/day, 7 days/week; 24 hours/ 7 days a week availability | Trained caregivers, nurses, LPNs, RNAs & companions | Greater Montreal | Fees (not specified) | Website available in FR, EN Service available in FR, EN, Italian, other languages | Not specified | Elderly, individuals suffering from a chronic or critical illness, caregivers & loved ones | Google |
| Soins Direct | Sole Proprietorship | In-Home Hospital | Respite care in the form of home care services including palliative care, post-operative care, care for the elderly, individuals with Alzheimer's, cognitive disorders, intellectual disabilities and/or pervasive developmental disorders; Palliative care includes pain/symptom management, personal hygiene, mobilization, meal preparation | Short periods of 3hrs | Nurses, LPNs & care attendants | Greater Montreal | Fees (not specified) | Website available in FR, EN | Not specified | Individuals with temporary or permanent loss of autonomy, caregivers & loved ones | Google |
| Humanika | Business Corporation or Company | In-Home CHSLD Hospital Private Residences | Respite consisting of home support services (shopping, meal preparation, light housekeeping), nursing care (including palliative care and hygiene care), activities, support & accompaniment | 24hrs/day, 7 days/week; Day, evening & night availability | PABs, social auxiliaries, companions, nurses, family physicians, occupational therapists, physiotherapists & notaries | Greater Montreal Lanaudière Laurentides, Montérégie Québec | Fees (32$/hour) | Website only available in FR Service available in FR, EN, Créole, Arabic | Not specified | Individuals with cognitive disorders (i.e. Alzheimer's), caregivers and loved ones All individuals with cognitive deficits (Alzheimer's, dementia,...), physical needs, surveillance, accompaniment, end-of-life care, palliative care & others | Google |
| Soins Tandem | Sole Proprietorship | In-Home CHSLD Hospital | Respite care consisting of supervision, reassuring presence, hygiene care, nutrition, assistance with exercises, transportation, palliative care & nursing care | 24hrs/day, 7 days/week ; Day, evening or night availability | Caregivers trained in CPR, PDSB, Loi 90 with DEP in assistance to the person | Centre-du-Québec Trois-Rivières | Fees (not specified) | Website only available in FR | Not specified | Individuals with a loss of autonomy, a disability, cognitive disorder, at the end-of-life, caregivers & loved ones | Google |
| Soli-Can Lac Saint-Jean Est | Non-Profit Organization | In-Home Outside the Home | Respite care consisting of supervision, assistance with nutrition/mobilization & activities; Hygiene care and other PAB tasks are not offered; Services offered to caregivers include counselling, massage, foot care, yoga, hypnosis & workshops | Few hours-Half day/week; Day availability only | Volunteers trained in palliative care, LPNs & PABs | Alma Saguenay-Lac-Saint-Jean | Free | Website only available in FR Service only available in FR | Individual in palliative care and/or caregivers Inability to travel | Individual in palliative care & their loved ones, caregivers | CCS L'Appui |
| Baluchon Répit Long Terme | Non-Profit Organization | In-Home | Respite in the form of assistance with daily activities (meal preparation, grocery shopping), hygiene, medication administration (except narcotics) & accompaniment following the patient's regular routine; Accomodation privileges & packages are offered to caregivers to help them recharge | Minimum of 4 days, up to 14 days per year; 24 hours/day, 7 days/week | Specialized & trained companion | Throughout Quebec | Fees (15$/day) | Website only available in FR Service available in FR, EN, Spanish | Alzheimer's disease or a related illness (in particular Lewy body dementia, vascular dementia or mixed dementia). Bedridden people  Parkinson's disease Amyotrophic lateral sclerosis Multiple sclerosis  Palliative care, end-of-life at home or other conditions | People with Parkinson's, ALS, MS, degenerative diseases or at the end-of-life | Google |
